# Supplementary material for: Klebsiella ARO112 promotes microbiota recovery, pathobiont clearance and prevents inflammation in IBD mice
Source: Nat Commun. 2025 Dec 11;16:10911. doi: 10.1038/s41467-025-67015-w (PMC12698716; doi:10.1038/s41467-025-67015-w)
Supplement: Supplementary file 1 — Supplementary Information [file 41467_2025_67015_MOESM1_ESM.pdf]

Supplementary Information

Supplementary Figure 1

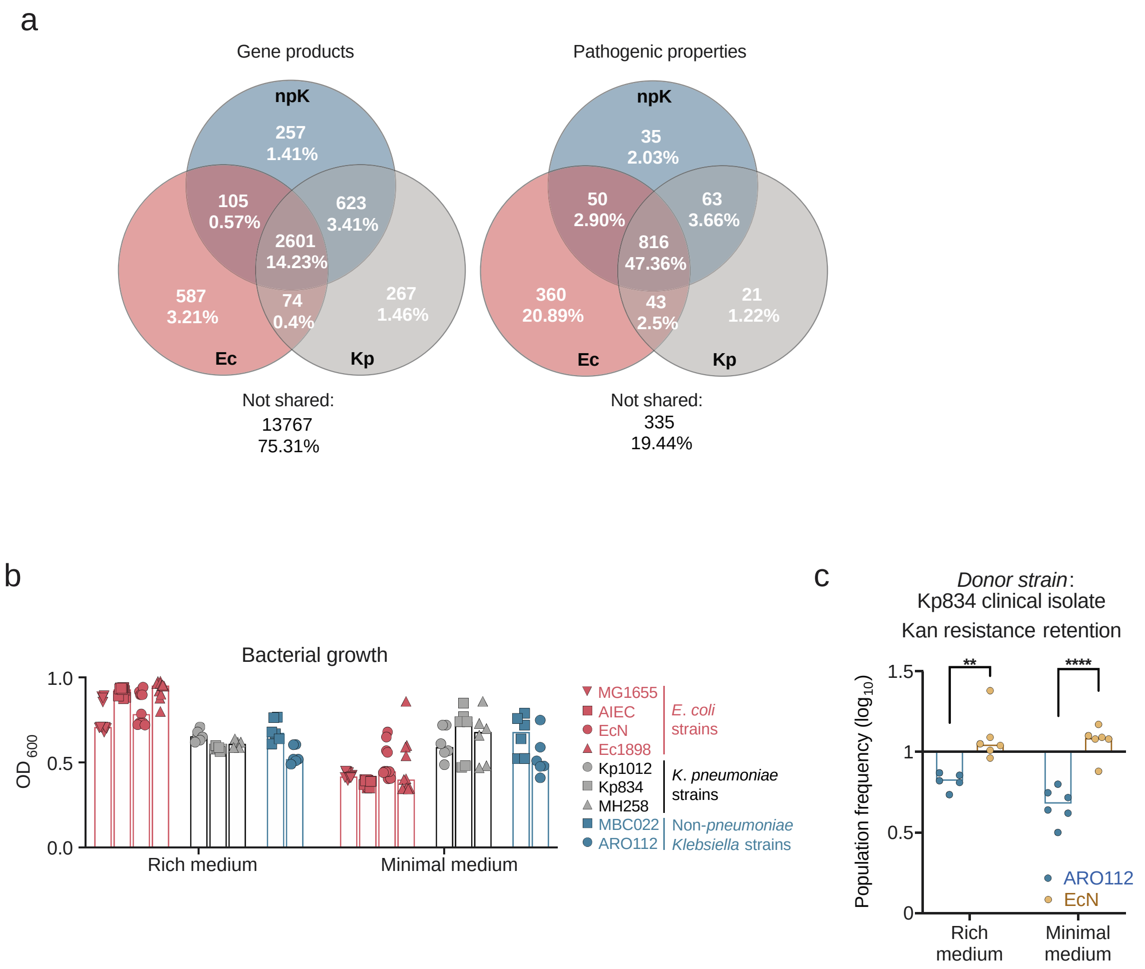

Supplementary Figure 1 | Comparison and correlations of phenotypically tested virulence traits. a, Venn diagrams of total gene products, shared by every member of each clade (left); Venn diagram of predicted pathogenic properties' hits shared by every member of each clade (right). Genome analyses were performed in PATRIC BV-BRC software, from where the number of hits for the different virulence factors databases were obtained. **b,** Bacterial growth in rich and minimal media. **c,** ARO112 and EcN were tested for their capacity to retain a natural antibiotic-resistant plasmid acquired, comparisons by Two-way ANOVA test with Sidak's correction for multiple comparisons (**p<0.01; **** p<0.0001). Growth was tested in 6-11 replicates, from 2 independent experiments. Natural conjugative plasmid retention was tested in a total of 5 replicates per group, from 2 independent experiments.

Supplementary Figure 2

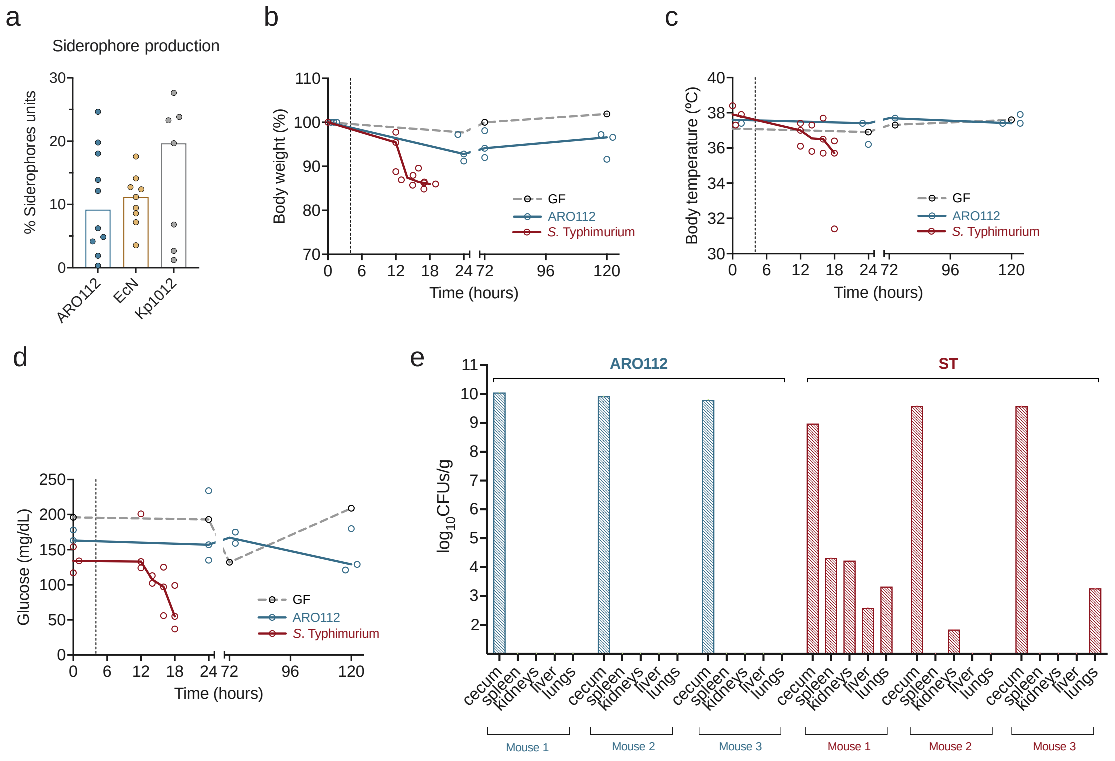

Supplementary Figure 2 | Siderophore production in mono-colonized mice, in vitro bacterial resistance to Lcn2, and in vivo experiments of mice mono-colonized with ARO112 or *S.* Typhimurium. a, Siderophores were measured in fecal supernatants collected from mice mono-colonized with each of selected strains (ARO112, EcN, Kp1012) for 5 days. Bars represent median values. **b-d**, germ-free and mono-colonized mice with ARO112 or *Salmonella* Typhimurium (ST) were monitored for: **b,** Body weight, **c,** body temperature, and **d,** blood glucose levels, at different time-points during colonization (0, 12. 14, 16, 18h for ST-colonized mice; 0, 24, 72, and 120h for germ-free and ARO112-colonized mice). **e,** Bacterial loads measured in the intestine (cecum) and translocated to extra-intestinal organs (spleen, kidneys, liver, and lungs). In **a**, siderophore production in fecal samples was tested in a total of 10 (ARO112), 9 (EcN), or 7 (Kp1012) samples, from at least 3 independent experiments. In **b-e** 3 mice colonized with ARO112, and 3 colonized with ST, and one germ-free as control, were tested.

Supplementary Figure 3

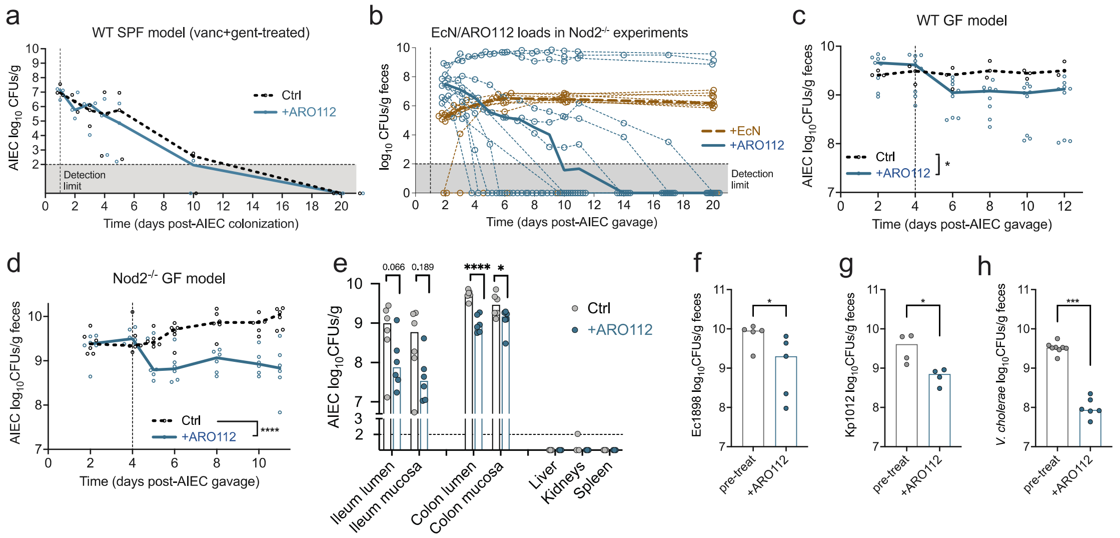

Supplementary Figure 3 | Probiotic colonization and evaluation of protective capacities of ARO112 and EcN against different invading bacteria in different mouse models. a, Loads of AIEC in fecal samples of WT mice treated with vancomycin and gentamicin and treated with PBS (Ctrl) or ARO112 probiotic (+ARO112). **b,** data from experiment shown in Figure 4, of the loads of ARO112 or EcN in fecal samples. **c,** Loads of AIEC in germ-free WT mice treated with PBS (Ctrl) or ARO112 probiotic (+ARO112). **d,** Loads of AIEC in germ-free Nod2$^{-/-}$ mice treated with PBS (Ctrl) or ARO112 probiotic (+ARO112) detected in feces and **e,** in samples from intestinal and extra-intestinal organs. Loads of **f,** Ec1898, **g,** Kp1012**,** and **h,** *V. cholerae* after 4 days of mono-colonization (pre-treat) or after 8 days of treatment with ARO112 (+ARO112), in germ-free WT mice. **i,** Loads of AIEC after 24h of growth without or with ARO112 or EcN, in rich or minimal media. AIEC loads in WT SPF models were tested in 3 replicates per group (**a**). Probiotic loads were tested in 12 (+ARO112) or 10 (+EcN) replicates from 2-3 independent experiments (**b**). AIEC loads in WT GF model were tested in 3 (Ctrl) and 9 (+ARO112) replicates in 2 independent experiments (**c**), while in Nod2$^{-/-}$ GF model were tested in 6 replicates per group in 2 independent experiments **(d)**, and data were analyzed using two-way ANOVA with Sidak's multiple comparison correction (* $p<0.05$, **** $p<0.0001$) (**c,d**). AIEC loads in intestinal and extra-intestinal organs in Nod2$^{-/-}$ GF model were measured in 6 and 3 replicates per group, respectively (**e**). Ec1898 loads were assessed in 5 replicates from 2 independent experiments (**f**). Kp1012 loads were assessed in 8 replicates from 2 independent experiments (**g**). *V. cholerae* loads were assessed from 6 replicates from 2 independent experiments (**h**). In **f-h**, data were analyzed using Mann-Whitney tests (* $p<0.05$; *** $p<0.001$). In **b**, thin lines represent ARO112 and EcN loads in individual mice over time, while in **a**, **b**, **c**, **d**, thick lines and bars represent median values.

Supplementary Figure 4

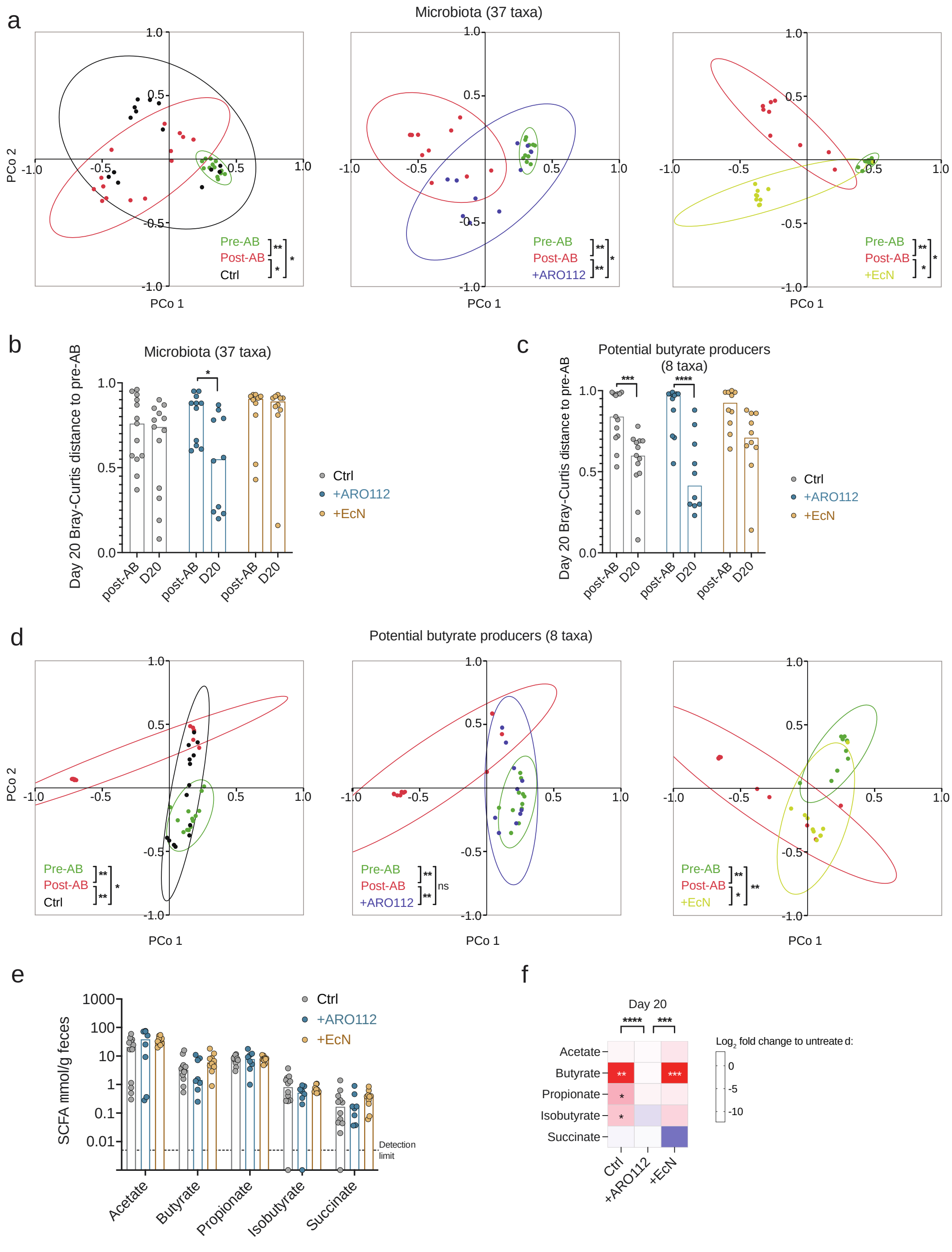

Supplementary Figure 4 | Comparison between treatments of different microbiota-related parameters in Nod2$^{-/-}$ mice after antibiotic treatment (Experimental setup shown in Fig. 4a). a, Principal Coordinate Analysis of Bray-Curtis dissimilarity index displaying clustering of samples before antibiotic treatment (pre-AB), after antibiotic treatment (post-AB), and after treatment (day 20 of experiment) with PBS (Ctrl; 14 samples) or probiotics (+ARO112 or +EcN; 10 samples per group), using microbiota composition (37 taxa). **b,** Analysis of the Bray-Curtis dissimilarity distances, obtained comparing the samples of each mouse pre-AB, to their position post-AB or post-treatment (day 20). **c,** Analysis of the Bray-Curtis dissimilarity distances, obtained comparing 8 taxa of potential butyrate

producers in the samples of each mouse pre-AB, to their position post-AB or post-treatment (day 20). **d,** Principal Coordinate Analysis of Bray-Curtis dissimilarity index displaying clustering of samples before antibiotic treatment (pre-AB), after antibiotic treatment (post-AB), and after treatment (day 5 of experiment) with PBS (Ctrl; 14 samples) or probiotics (+ARO112 or +EcN; 10 samples per group), for the 8 taxa of potential butyrate producers depleted by antibiotics (*Alistipes*, *Syntrophococcus*, *Ruminococcus*, *Butyricicoccus*, *Dorea*, *Oscillibacter*, unclassified Ruminococcacea, unclassified Lachnospiraceae). **e,** Fecal levels of SCFA measured from untreated mice (day -4). **f,** Heatmap displaying the fold change of SCFA levels at day 20 compared to untreated levels. Ellipses in **a** and **d** represent 90% confidence intervals. One-way PERMANOVA with 999 permutations were performed with Bonferroni corrections (**a** and **d**) and two-way ANOVA were tested with Sidak's correction for multiple comparisons (**b** and **c**) (* $p<0.05$; ** $p<0.01$; *** $p<0.001$; **** $p<0.0001$).

Supplementary Figure 5

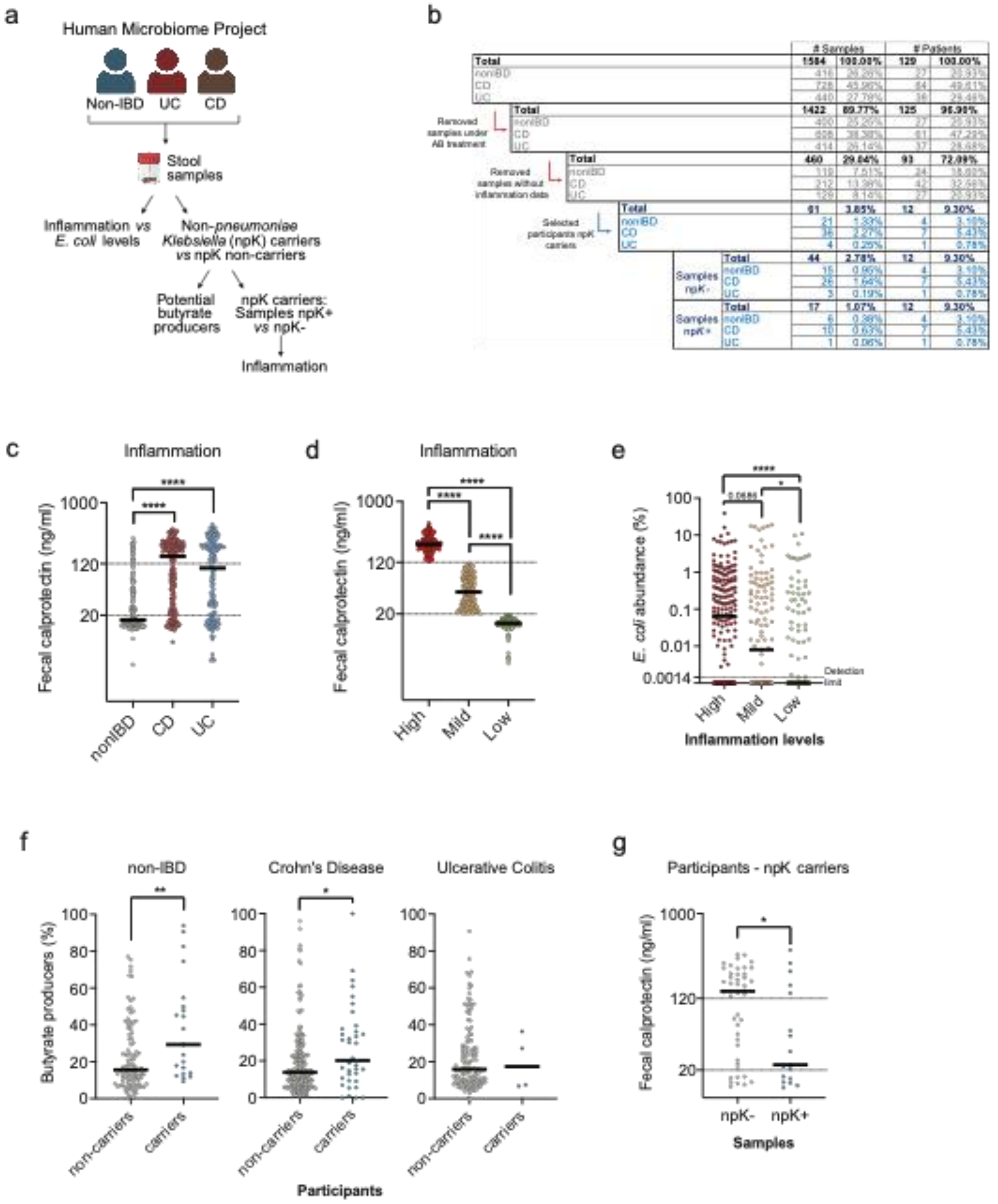

Supplementary Figure 5| Abundance of non-*pneumoniae Klebsiella* species correlate with higher levels of potential butyrate producers and lower levels of inflammation in human fecal samples. a, Cohort details. Specific microbiota members and inflammation levels were analyzed in samples from non-IBD, UC, and CD patients of the HMP2. **b,** Table with sorting pipeline for samples from human patients. **c,** Levels of fecal calprotectin in patients with IBD (CD and UC) and non-IBD participants. **d,** Human samples display three distinct levels of fecal calprotectin: very high (>120 ng/ml), high (>20 ng/ml), and low (<20 ng/ml). **e**, *E. coli* abundance in samples with high (>120 ng/ml), mild (>20 ng/ml), and low (<20 ng/ml) levels of fecal calprotectin. **f,** Abundance of potential butyrate producer families (Lachnospiraceae, Oscillispiraceae, Ruminococcaceae) in carriers or non-carriers of non-*pneumoniae Klebsiella* species (npK) in non-IBD participants or patients with CD or UC. **g,** Fecal calprotectin levels in carriers (participants with at least one positive sample for non-*pneumoniae Klebsiella spp*) in samples from these individuals with or without detectable levels of npK. Fecal

calprotectin was tested in 119 (non-IBD), 212 (CD), 129 (UC) (c), and 192 (high), 127 (mild), and 141 (low). (**c,d**) samples, using the Kruskal-Wallis test corrected for multiple comparisons by Dunn's test. In **e-g**, lines represent median values. *E. coli* abundance was tested in 192 (high), 127 (mild), and 141 (low) samples, were compared using the Kruskal-Wallis test corrected for multiple comparisons with Dunn's test (**e**). Abundance of potential butyrate producers in 98 (non-IBD non-carriers), 21 (non-IBD carriers), 176 (CD non-carriers), 36 (CD carriers), 125 (UC non-carriers), and 4 (UC carriers) samples, was compared using the Mann-Whitney test (**f**). Inflammation levels were tested in 44 (npK-) and 17 (npK+) samples, using the Mann-Whitney test (**g**). * $p<0.1$; ** $p<0.01$; **** $p<0.0001$. Panel a was adapted from a figure created in BioRender. Oliveira, R. (2025) https://BioRender.com/qnvxb7c.

Supplementary Figure 6

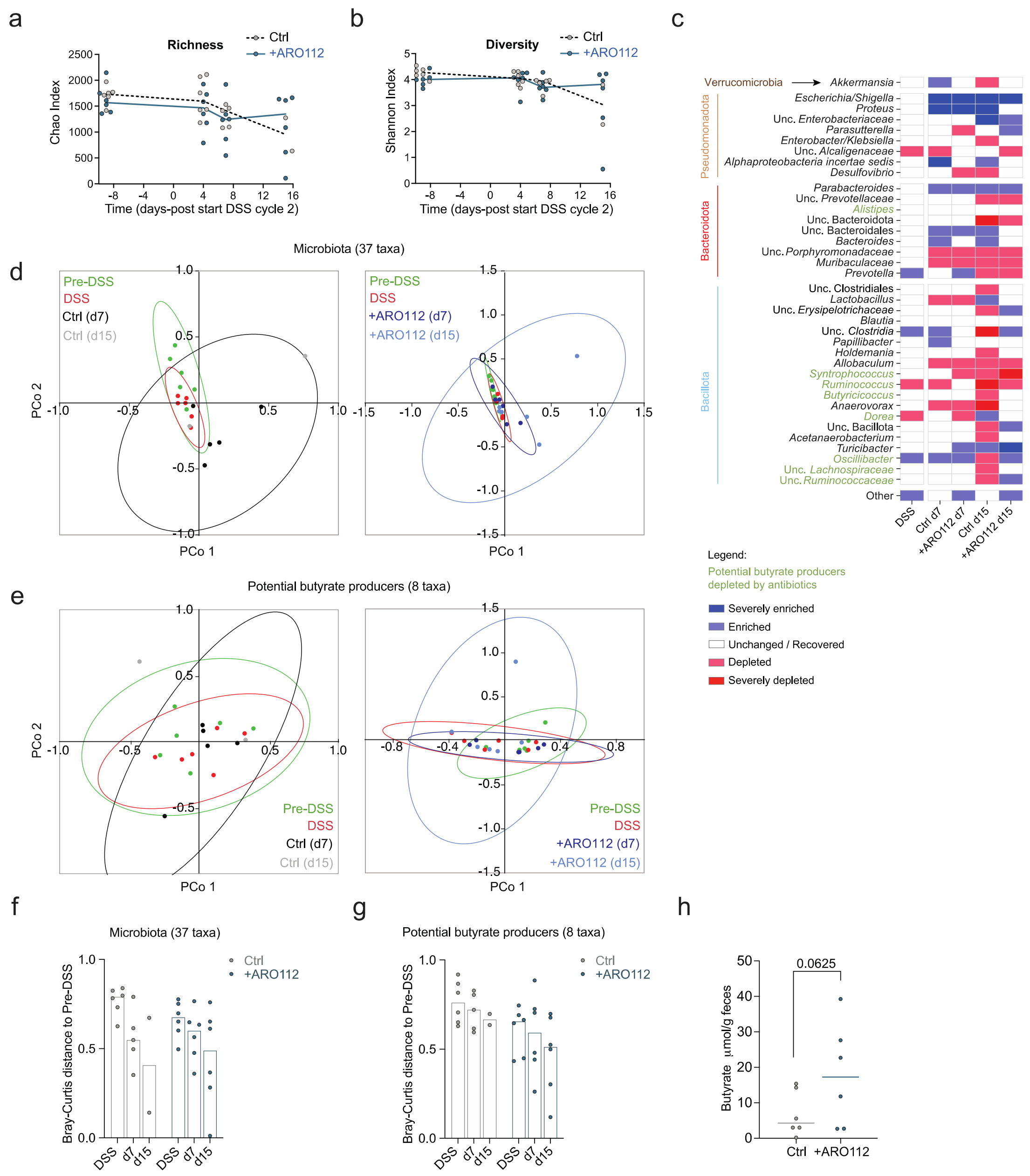

Supplementary Figure 6 | Comparison between treatments of different microbiota-related parameters in experiment in Nod2$^{-/-}$ mice treated with DSS (experimental setup shown in Fig. 7). a, Richness and **b,** diversity of gut microbiota in fecal samples collected before (pre-DSS, day -9), or during the second DSS cycle (DSS, days 4 and 7) and 7 days post-DSS (day 15) of mice untreated (Ctrl) or treated with ARO112. In **a** and **b**, lines represent median values for each treatment over time. **c,** Changes in the relative abundances of most prevalent taxa at days 4, 7 and 15 post-DSS cycle in comparison with the levels pre-DSS (day -9). **d,** Principal Coordinate Analysis of Bray-Curtis dissimilarity index displaying clustering of samples before DSS (pre-DSS), during DSS (DSS), and after treatment (days 7 and 15 of experiment) with PBS (Ctrl; 5-6 samples) or +ARO112 (6 samples), using microbiota composition (37 taxa). **e,** Principal Coordinate Analysis of Bray-Curtis dissimilarity index displaying clustering of samples before DSS (pre-DSS), during DSS (DSS), and after treatment (days 7 and 15 of experiment) with PBS (Ctrl; 5-6 samples) or +ARO112 (6 samples), for the 8 taxa of potential

butyrate producers depleted by antibiotics (*Alistipes*, *Syntrophococcus*, *Ruminococcus*, *Butyricicoccus*, *Dorea*, *Oscillibacter*, unclassified Ruminococcacea, unclassified Lachnospiraceae). **f,** Analysis of the Bray-Curtis dissimilarity distances, obtained comparing the samples of each mouse pre-DSS, to their position post-DSS or post-treatment (days 7 or 15 of experiment), using microbiota composition (37 taxa). **g,** Analysis of the Bray-Curtis dissimilarity distances, obtained comparing the samples of each mouse pre-DSS, to their position post-DSS or post-treatment (days 7 or 15 of experiment), for the 8 taxa of potential butyrate producers depleted by antibiotics (*Alistipes*, *Syntrophococcus*, *Ruminococcus*, *Butyricicoccus*, *Dorea*, *Oscillibacter*, unclassified Ruminococcacea, unclassified Lachnospiraceae). **h,** Fecal levels of butyrate measured at day 7 of experiment. In **a**-**c**, data were analyzed using Two-way ANOVA with Dunnett's correction for multiple comparisons (* $p<0.1$; ** $p<0.01$; *** $p<0.001$; **** $p<0.0001$). Fecal microbiota composition analyses were performed in a total of 14 (Ctrl), 10 (+ARO112), or 4-10 (+EcN) samples, from 2-4 independent experiments. Ellipses in **d** and **e** represent 90% confidence intervals. One-way PERMANOVA with 999 permutations were performed with Bonferroni corrections (**d** and **e**) and Wilcoxon test was performed (**h**).

Supplementary Figure 7

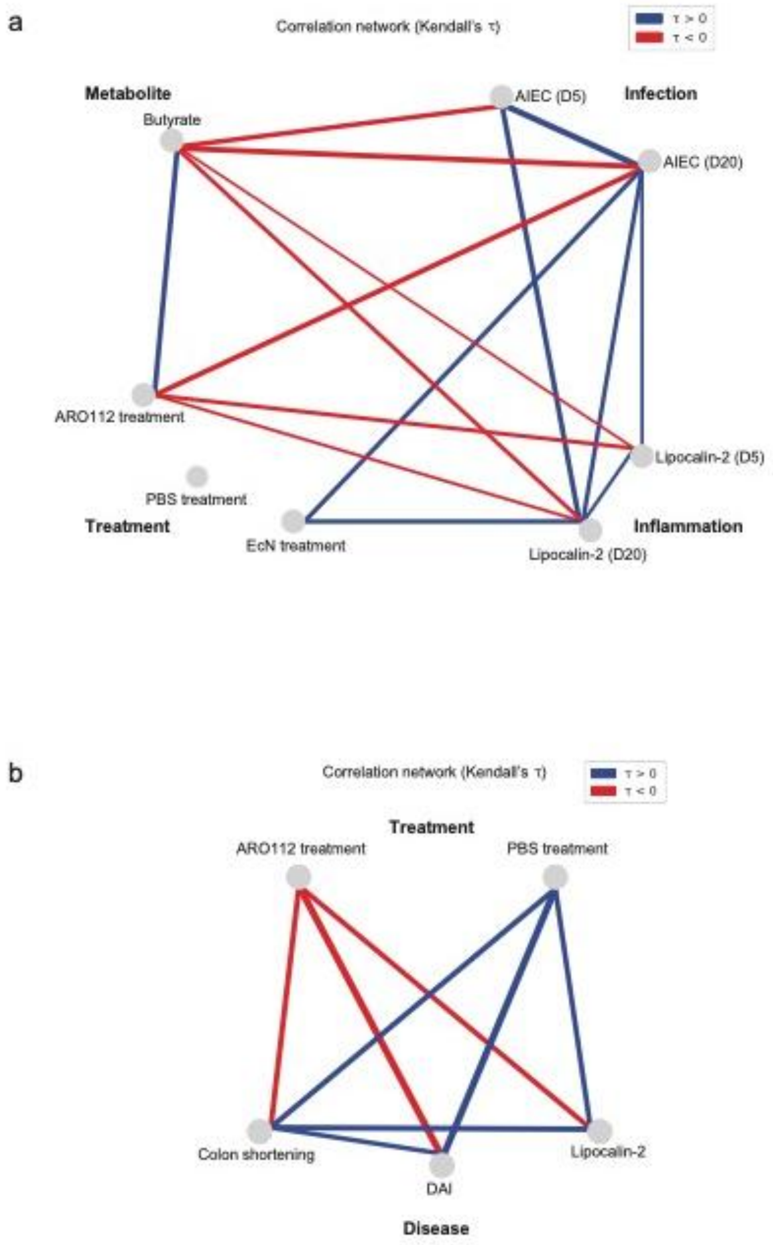

Supplementary Figure 7 | Kendall's Tau correlation networks between probiotic treatments and health and disease markers. Kendall's Tau network showing positive (blue edges) and negative (red edges) correlations between experimental measurements and parameters. **a,** Infections (AIEC at days 5 and 20), and PBS, ARO112, and EcN treatments are binary, while butyrate and Lipocalin-2 levels are continuous data. **b,** PBS and ARO112 treatments are binary, disease activity index (DAI) is the average of disease activity score during the experiment, Lipocalin-2 levels at day 7 and colon shortening (1/colon length at day 7) are continuous data. Two-tailed Kendall's τ rank correlation. Only correlations with $\tau>0.2$ or $\tau<-0.2$ and significant ($p<0.05$) are depicted. Correlations between treatments were ignored and not represented.
