# Supplementary material for: Klebsiella ARO112 promotes microbiota recovery, pathobiont clearance and prevents inflammation in IBD mice
Source: Nat Commun. 2025 Dec 11;16:10911. doi: 10.1038/s41467-025-67015-w (PMC12698716; doi:10.1038/s41467-025-67015-w)
Supplement: Supplementary file 2 — Description of Additional Supplementary Files [file 41467_2025_67015_MOESM2_ESM.pdf]

Title: Supp. Data 1

Description: Strain and genome list used in this study.

Title: Supp. Data 2

Description: Presence and absence of pathogenic properties in Enterobacteriaceae genomes tested, according to virulence factors databases.

Title: Supp. Data 3

Description: Presence and absence of gene products annotated as conjugation-, competence-, multidrug-, transposase-, phage/integrase-, and CRISPR-related in Enterobacteriaceae genomes tested.

Title: Supp. Data 4

Description: Abundance (gene counts) of pathogenic properties in Enterobacteriaceae genomes tested, according to virulence factors databases.

Title: Supp. Data 5

Description: List of pathogenic traits that drive PCoA separation.

Title: Supp. Data 6

Description: Disease Activity Index scores and subscores (weight loss, stool consistency, bleeding).

Title: Supp. Data 7

Description: Parameters of metabolite (butyrate concentration), infection (AIEC loads), and inflammation (Lipocalin-2 levels) measured in the AB-treated mice and used for the interaction network analysis.

Title: Supp. Data 8

Description: Parameters of disease (colon shortening, disease activity score, and Lipocalin-2 levels) measured in the DSS-treated mice and used for the interaction network analysis.

Title: Supp. Data 9

Description: In vitro and in vivo measurements of parameters (biofilms, human serum resistance, urease activity, siderophore production, AIEC CFUs, SCFA, Lipocalin-2)

Title: Supp. Data 10

Description: Compilation of selected data from the Human Microbiome Project (HMP2), publicly available at https://www.ibdmdb.org
