# Supplementary material for: Klebsiella ARO112 promotes microbiota recovery, pathobiont clearance and prevents inflammation in IBD mice
Source: Nat Commun. 2025 Dec 11;16:10911. doi: 10.1038/s41467-025-67015-w (PMC12698716; doi:10.1038/s41467-025-67015-w)
Supplement: Supplementary file 13 — Reporting Summary [file 41467_2025_67015_MOESM13_ESM.pdf]

Corresponding author(s): Vitor Cabral, Rita A. Oliveira, Karina B. Xavier

Last updated by author(s): Nov 13, 2025

Reporting Summary

Nature Portfolio wishes to improve the reproducibility of the work that we publish. This form provides structure for consistency and transparency in reporting. For further information on Nature Portfolio policies, see our Editorial Policies and the Editorial Policy Checklist.

Statistics

For all statistical analyses, confirm that the following items are present in the figure legend, table legend, main text, or Methods section.

n/a	Confirmed	
☐	☒	The exact sample size (*n*) for each experimental group/condition, given as a discrete number and unit of measurement
☐	☒	A statement on whether measurements were taken from distinct samples or whether the same sample was measured repeatedly
☐	☒	The statistical test(s) used AND whether they are one- or two-sided<br>*Only common tests should be described solely by name; describe more complex techniques in the Methods section.*
☒	☐	A description of all covariates tested
☐	☒	A description of any assumptions or corrections, such as tests of normality and adjustment for multiple comparisons
☐	☒	A full description of the statistical parameters including central tendency (e.g. means) or other basic estimates (e.g. regression coefficient) AND variation (e.g. standard deviation) or associated estimates of uncertainty (e.g. confidence intervals)
☐	☒	For null hypothesis testing, the test statistic (e.g. *F*, *t*, *r*) with confidence intervals, effect sizes, degrees of freedom and *P* value noted<br>*Give P values as exact values whenever suitable.*
☒	☐	For Bayesian analysis, information on the choice of priors and Markov chain Monte Carlo settings
☒	☐	For hierarchical and complex designs, identification of the appropriate level for tests and full reporting of outcomes
☒	☐	Estimates of effect sizes (e.g. Cohen's *d*, Pearson's *r*), indicating how they were calculated

Our web collection on statistics for biologists contains articles on many of the points above.

Software and code

Policy information about availability of computer code

Data collection	Bruker988 NEO 500MHz instrument equipped with QXI H-C/N/P 5 mm probe-head with z-gradients was used for NMR data collection. Multiskan Sky plate reader was used to measure optical densities. Illumina miSeq was used to sequence whole genomes and 16S data. NGS and 16s data generated in this study were deposited in the European Nucleotide Archive (ENA) under the accession code PRJEB102263.
Data analysis	Mothur v.1.32.1 was used to process sequences, while ChimeraSlayer was used to remove potential chimeric sequences. BV-BRC browser software (v3.29.20) was used to build a phylogenetic tree, using Multiple Alignment using Fast Fourier (MAFFT) Transform alignment program and RAxML Fast Bootstrapping branch support method (v8.2.11). Phylogenetic tree (Figure 1a) was done using iTOL (itol.embl.de). Cluster heatmap (Figure 1b) was done using SRPlot (https://www.bioinformatics.com.cn), using bidirectional clustering, complete cluster method and Euclidean distances. Networks of interactions (Supplementary Figure 7) were computed with networkx v3.3 and rendered by matplotlib v3.10.0, using the Jupyter notebook v7.2.2 running Python v3.12.3, including pandas v2.2.3, numpy v1.26.4, and scipy v1.13.1 packages. Principal Coordinate Analyses were obtained with Past4 v02 software (https://folk.universitetetioslo.no/ohammer/past) as explained above. Schematic diagrams were generated in BioRender and figures were assembled and adapted using Adobe Illustrator 2025 v29.8.2.

For manuscripts utilizing custom algorithms or software that are central to the research but not yet described in published literature, software must be made available to editors and reviewers. We strongly encourage code deposition in a community repository (e.g. GitHub). See the Nature Portfolio guidelines for submitting code & software for further information.

Data

Policy information about availability of data

All manuscripts must include a data availability statement. This statement should provide the following information, where applicable:

- Accession codes, unique identifiers, or web links for publicly available datasets
- A description of any restrictions on data availability
- For clinical datasets or third party data, please ensure that the statement adheres to our policy

The NGS data generated in this study have been deposited in the ENA (European Nucleotide Archive) database under the study accession code PRJEB102263. All other data generated in this study are provided in the Supplementary Information/Supplementary Dataset files.

Research involving human participants, their data, or biological material

Policy information about studies with human participants or human data. See also policy information about sex, gender (identity/presentation), and sexual orientation and race, ethnicity and racism.

Reporting on sex and gender	This study used publicly available, de-identified human data obtained from the Inflammatory Bowel Disease Multi'omics Database (IBDMDB; https://ibdmdb.org/). No new data were collected from human participants, and no identifiable information was accessed. All data were originally collected under appropriate institutional ethics approvals and participant consent by the IBDMDB consortium. Sex and gender information were not analyzed or reported in this study. The publicly available dataset used (IBDMDB) includes de-identified participant metadata, but our analyses did not stratify by sex or gender.
Reporting on race, ethnicity, or other socially relevant groupings	Race, ethnicity, or other socially defined groupings were not analyzed or reported. The IBDMDB dataset contains de-identified participant data and was not used here to explore demographic variables.
Population characteristics	The analyzed data originate from human participants enrolled in the IBDMDB cohort, including individuals with and without inflammatory bowel disease, as described in the original study (Lloyd-Price et al., Nature, 2019). Our analyses focused solely on fecal calprotectin values and microbiota composition metrics, without further demographic or clinical stratification.
Recruitment	No participant recruitment was performed for this study. All data were obtained from the publicly available IBDMDB resource, where recruitment procedures are detailed in the original publications.
Ethics oversight	Ethical approval and participant consent for the collection and use of IBDMDB data were obtained by the original investigators, as described in Lloyd-Price et al., Nature, 2019.As this work used only publicly available, de-identified data, no additional ethics approval was required for this secondary analysis.

Note that full information on the approval of the study protocol must also be provided in the manuscript.

Field-specific reporting

Please select the one below that is the best fit for your research. If you are not sure, read the appropriate sections before making your selection.

☒ Life sciences ☐ Behavioural & social sciences ☐ Ecological, evolutionary & environmental sciences

For a reference copy of the document with all sections, see nature.com/documents/nr-reporting-summary-flat.pdf

Life sciences study design

All studies must disclose on these points even when the disclosure is negative.

Sample size	From 3--15 mice were used in each group. Sample size was chosen according to institutional directives and in accordance with the 3Rs rules (Replacement, Reduction and Refinement) guiding principles underpinning the humane use of animals in research, but no statistical analyses were performed to predetermine the sample sizes. N=3 is the minimum number of independent subjects to be used for statistical analysis to be performed.
Data exclusions	No data were excluded.
Replication	All attempts at replication were successful, with multiple mice in each group (see sample size above).All experiments were done at least twice, with the exception of in vivo experiment with WT mice treated with vancomycin and gentamicin, and gnotobiotic experiment with Kp1012, which were done only once.
Randomization	Mice were randomly allocated to different treatments. We ensured that in each experiment all mice were siblings and shared the same cage for microbiome homogenization prior to the experiment.

Blinding	Blinding was used for histological scoring and NMR data acquisition and quantification.

Reporting for specific materials, systems and methods

We require information from authors about some types of materials, experimental systems and methods used in many studies. Here, indicate whether each material, system or method listed is relevant to your study. If you are not sure if a list item applies to your research, read the appropriate section before selecting a response.

Materials & experimental systems

n/a	Involved in the study
☒	☐ Antibodies
☒	☐ Eukaryotic cell lines
☒	☐ Palaeontology and archaeology
☐	☒ Animals and other organisms
☒	☐ Clinical data
☒	☐ Dual use research of concern
☒	☐ Plants

Methods

n/a	Involved in the study
☒	☐ ChIP-seq
☒	☐ Flow cytometry
☒	☐ MRI-based neuroimaging

Animals and other research organisms

Policy information about studies involving animals; ARRIVE guidelines recommended for reporting animal research, and Sex and Gender in Research

Laboratory animals	All mice (Mus musculus) used in this study were supplied by the Rodent Facility at GIMM and were given ad libitum access to food (Rat and Mouse No.3 Breeding – Special Diets Services, product number 801030) and water. Mice were kept at 20-24 °C and 40-60% humidity with a 12-h light-dark cycle.<br>C57BL/6J mice, either WT or Nod2-/-, were used at 6–24 weeks of age and were randomly assigned to experimental and control groups and kept singly caged throughout the experiment. C57BL/6J mice were used for all experiments. None of the animal experiments were performed blinded. Sample size was chosen according to institutional directives and in accordance with the guiding principles underpinning humane use of animals in research. No statistical analyses were performed to predetermine the sample sizes. All of the experiments were performed at least twice, except when stated otherwise.
Wild animals	No wild animals were used.
Reporting on sex	Both males and females were used in experiments.
Field-collected samples	This study did not involve samples collected from the field.
Ethics oversight	All experiments performed with C57BL/6 mice were included in an animal experimentation protocol approved by IGC ethics committee and the Portuguese National Entity (DGAV) - Protocol number A003.2022

Note that full information on the approval of the study protocol must also be provided in the manuscript.

Plants

Seed stocks	*Report on the source of all seed stocks or other plant material used. If applicable, state the seed stock centre and catalogue number. If plant specimens were collected from the field, describe the collection location, date and sampling procedures.*
Novel plant genotypes	*Describe the methods by which all novel plant genotypes were produced. This includes those generated by transgenic approaches, gene editing, chemical/radiation-based mutagenesis and hybridization. For transgenic lines, describe the transformation method, the number of independent lines analyzed and the generation upon which experiments were performed. For gene-edited lines, describe the editor used, the endogenous sequence targeted for editing, the targeting guide RNA sequence (if applicable) and how the editor was applied.*
Authentication	*Describe any authentication procedures for each seed stock used or novel genotype generated. Describe any experiments used to assess the effect of a mutation and, where applicable, how potential secondary effects (e.g. second site T-DNA insertions, mosiacism, off-target gene editing) were examined.*
